# A CMAC-based scheme for determining membership with classification of text strings

**DOI:** 10.1007/s00521-015-1989-6

**Published:** 2015-07-10

**Authors:** Heng Ma, Ying-Chih Tseng, Lu-I. Chen

**Affiliations:** 1 Department of Industrial Management, Chung Hua University, No. 707, Sec.2, WuFu Rd., Hsinchu, Taiwan; 2 Ph.D. Program of Technology Management, Chung Hua University, Hsinchu, Taiwan

**Keywords:** Membership determination, Classification, Text string, Neural network

## Abstract

Membership determination of text strings has been an important procedure for analyzing textual data of a tremendous amount, especially when time is a crucial factor. Bloom filter has been a well-known approach for dealing with such a problem because of its succinct structure and simple determination procedure. As determination of membership with classification is becoming increasingly desirable, parallel Bloom filters are often implemented for facilitating the additional classification requirement. The parallel Bloom filters, however, tend to produce additional false-positive errors since membership determination must be performed on each of the parallel layers. We propose a scheme based on CMAC, a neural network mapping, which only requires a single-layer calculation to simultaneously obtain information of both the membership and classification. A hash function specifically designed for text strings is also proposed. The proposed scheme could effectively reduce false-positive errors by converging the range of membership acceptance to the minimum for each class during the neural network mapping. Simulation results show that the proposed scheme committed significantly less errors than the benchmark, parallel Bloom filters, with limited and identical memory usage at different classification levels.

## Introduction

Text strings are widely used as identifiers in our daily lives, such as Internet access accounts and passwords, email addresses, car license plates and credit cards, which are also employed for coding parts, processes and products in manufacturing systems as well as service industries. Such identifiers are inevitably of great amounts since they are used for the identification purpose and therefore identical codes are not allowed. When a group of identifiers is associated with a certain characteristic, it becomes imperative to find out whether a random identifier belongs to this group for some applications. For example, it is often imminent to determine whether a car license plate is actually a stolen car or whether an email address is a source of commercial advertisements. In this paper, membership refers to the status of whether a random identifier exists in a group with a certain characteristic. Membership determination is an important procedure when a text string identifier is required to be a legitimate member of the characteristic group, also referred to as the payload. The procedure, however, could be a time-intensive task as the payload is growing larger and larger, which is especially significant when exact string matching methods [1] are utilized, since they usually require a great deal of primary memory space for storing the payload in a certain fashion [2] or constructing auxiliary indexing mechanisms [3] for the searching purposes. Therefore, it is considered as an impractical means to perform string matching for checking membership because the computational complexity still depends on the size of payload.

**Fig. 1:**
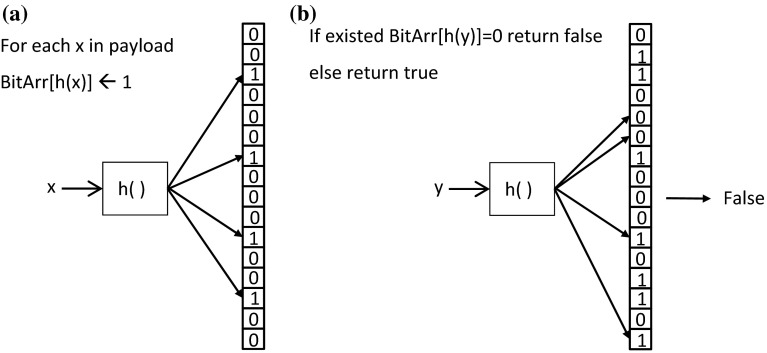
Illustration of Bloom filter with pseudocodes. **a** Programming phase, **b** checking phase

Bloom filter [4] has been successful in applications requiring membership checking [5, 6] because a nearly constant processing time could be achieved regardless of the size of payload. The Bloom filter approach involves a hashing process for transforming input strings into addresses on a bit array, also referred to as the programming phase, and a simple determination rule for checking membership (i.e., the checking phase). In the programming phase, all bits in the array are initially set to 0 and all the bits addressed by the hashing process for all the strings in the payload are turned to 1. The bit value remains 1 when it is addressed by several strings. With such a process, it can be realized that there are no false-negative errors in the checking phase after the programming phase is completed because a string is only considered as a member when all the bits addressed by the string are 1. Figure 1 shows the illustration with pseudocodes for both phases of Bloom filter. False positives, however, are possible because a non-member string could address to all the bits whose values are 1. Such a situation is particularly significant when the size of the bit array is insufficient, resulting in a high ratio of “1” bits in the array, and thus, false positives could often occur.

During the past decade, determination of membership with classification or attribute has drawn researchers’ attention [7, 8] since it becomes increasingly desirable when membership could accompany with class information simultaneously. Normally, the class could represent a number of basic attributes or features associated with that string identifier, such as a car’s build, make and color with the license plate numbers and someone’s usage history with his/her email addresses. The classes could be in an integer form for one particular attribute or a combination of multiple attributes. Membership with class determination could greatly reduce additional procedures and bandwidth for accessing database on the service machine for retrieving those attributes of interest once the membership is identified as positive. In the literature, parallel Bloom filters (PBFs) were often implemented to deal with this problem [9, 10], where each layer represents a different class and membership checking must be performed on all the parallel layers. When only one layer is identified as positive membership, the associated attributes assigned to that layer are also recognized. However, since the Bloom filters are inherently accompanied with a certain rate of false-positive errors, as analyzed in [11], the rate could be multiplied with the parallel architecture because a query string must pass through all the layers for checking its membership. In [12], the hierarchical Bloom filters (HBFs) were developed for characterizing payload attribution in network environments. In a sense, the HBF structure is composed of a number of blocked Bloom filters that could provide probabilistic answers to membership queries on the excerpts of payloads; nevertheless, multilayer calculations for membership decision are still required.

More recently, efforts have been made in an attempt to optimize key performance criteria of Bloom filter, such as query overhead, memory usage and false-positive ratio, by using alternatives of the bit array or designing specific data structures. For example, the binary bits were extended to state symbols for implementing a state machine for dynamic flows in networking [16]. Besides state symbols, multi-bit words were also employed as an auxiliary array, where membership could be determined in only a small number of memory accesses [21]. Taking reducing memory access into account, a collision-free hashing scheme was developed on discriminative Bloom filters to speed up the lookup process [20]. A combinatorial Bloom filter was proposed using multiple sets of hash functions to code an input element into a binary array as the group identity of the element. This approach, utilizing only a single-bit array, is capable of achieving membership determination with classification by using a considerable amount of hash functions [19]. Classification is so important in network applications; in [17], multiple Bloom filters were implemented as a packet classifier, where a multitier configuration was proposed to increase the throughput. As far as lowering false positives is concerned, it has been recognized as a trade-off between the size of the bit array and the false-positive ratio. A locality-sensitive Bloom filter was proposed, where the hashing functions were configured accordingly for improving the false-positive ratio of approximate membership query [18]. An architecture for reducing the false-positive rate was proposed in [22], where a great number of false positives on the main Bloom filter in a reasonable size can be recognized by the implementation of the cross-checking Bloom filters.

To avoid multilayer membership checking for determining membership with classification and meet the performance criteria, we propose a scheme based on a neural network mapping known as the cerebellar model articulation controller (CMAC) [13], which can provide membership and classification information with a single-layer calculation. CMAC was first developed for controlling robotic manipulators, which was later recognized as a neural network paradigm due to its capabilities of learning and generalization. This particular type of neural network mapping comprises an associative array of real numbers, while the Bloom filter is composed of bit values in a similar layer. Because of the real numbers, different target values could be assigned as class identification for the membership-checking purpose. CMAC has been successfully applied on a variety of fields, such as control, signal processing and networking [14]. The generalization capability of CMAC was originally achieved by a so-called concatenation method, which replaces the prefixes of strings by sequential alphabetic characters for coding into the addresses in the associative array. In our implementation, the concatenation method is disregarded because the generalization is no longer required as far as membership determination is concerned. We employ a mapping process similar to the Bloom filters, where a hash function is specifically designed for text strings. The proposed hash function is used for simulations of both the PBFs and the proposed CMAC-based scheme throughout this paper.

## Hash function for text string

In the original form of the Bloom filter, multiple hash functions were incorporated for mapping input elements to the associative array, each of which is responsible for addressing one cell in the array. Such a mapping agenda works well; however, there are no rules for deciding the number and type of the hash functions utilized. Furthermore, multiple hash functions also cause additional computational overhead. As pointed out in [9], a single hash function could also be suitable for the mapping purpose as long as the uniformity of cell addressing in the associative layer is sustained. To develop a suitable hash function for text strings, we propose that each character in a text string addresses one cell in the associative layer. The uniformity could be achieved by taking advantage of the character’s designated code and its position in the string identifier. We adopt the logarithm function as proposed in [15], where the randomness is achieved by discarding the integral part and the first few digits of the decimal part of the outcome from the logarithm function as in (1).

1 $$h\left( x \right) = D\left( {\log \left( x \right) \times 10^{c} } \right)$$

In (1), *h*(*x*) is the hash function of a positive number *x*, *D*(·) is the decimal part of the content, *c* is the number of digits to be discarded in the decimal part after the logarithm function is applied. We used *c* = 4 in this paper.

There are two steps in the proposed hash function: calculating the starting position in the associative layer for an input string as in (2) and sequentially finding the address for each character of the input string as in (3). The notation used in this paper is as follows: *s* designates an arbitrary string, *s*(*k*) is the *k*th character of *s*, *s*_*i*_ is the *i*th string in the payload, *s*_*i*_(*k*) is the *k*th character of *s*_*i*_.

2 $$p\left( s \right) = I\left( {h\left( {\mathop \sum \limits_{k = 1}^{l\left( s \right)} s\left( k \right) \times k} \right) \times M} \right) + 1$$

3 $$a\left( {s\left( k \right)} \right) = p\left( s \right) + I\left( {h\left( {\mathop \sum \limits_{j = 1}^{k} s\left( j \right) \times j} \right)} \right)\% M + 1 \quad k = 1\sim\;l\left( s \right)$$

In (2), *p*(*s*) is the starting position of the associative layer for string *s*, *l*(*s*) is the length of *s*, *s*(*k*) is the *k*th character of *s*, *M* is the size of the associative layer. We used ASCII codes for *s*(*k*) in this paper. The address index of the cells in the associative layer is 1 to *M*. In (3), *a*(*s*(*k*)) is the address of *s*(*k*) in the associate layer, *I*(·) denotes the integral part, % is the operator for calculating the remainder divided by *M*. In a sense, (2) is to avoid the situation where only a portion of the associative layer is addressed because sometimes there is a high similarity of the text strings in the payload. To differentiate a character’s designated code and its position in the string, we multiple each character by its position number in a cumulated fashion as in (3). We employed the $\chi^{2}$ test to examine the uniformity of addressed cells in the associative layer for the proposed hash function. The test data were randomly generated and composed of legitimate characters with an ACSII code. As a result, there was no significant evidence to deny the uniformity at the 5 % confidence level. The proposed hash function for text strings is convenient and easy to use because only the logarithm function is required. The uniformity is achieved by taking advantage of each character’s designated code and position in the string.

## Parallel Bloom filters versus proposed scheme

In a way, PBFs and the proposed scheme are very similar in their two-phase operations, i.e., a set of text strings must be transformed to a vector of associative array before they can be used as a checking mechanism for membership and classification. For PBF, such transformation is referred to as “programming,” while “learning” is used for the proposed scheme since it requires a number of iterations of payload presentation for adjusting the array vector. In this paper, we use “programming” for both schemes. The second phase, referred to as “checking,” is responsible for determining membership and classification of a query string using the associative arrays resulted from the first phase. Normally, the programming or learning phase is performed in an off-line mode for obtaining a suitable array vector, while the checking phase is an on-line operation that takes query strings and then provides prompt responses.

### The parallel Bloom filters

In the programming phase of PBF, several transformations must be made depending on the number of classes or subsets in the payload as in (4). Initially, all cells of the parallel layers are set to 0. Each text string in a subset is sequentially presented to the hash function, whose outcomes would turn all the addressed cells of the corresponding layer to 1. The phase stops when all the strings in the payload are presented to the transformation process.

4 $$\left\{ {\text{PL}} \right\}_{i} \to \varvec{A}_{i} \quad i = 1\sim\;n$$

where $\left\{ {\text{PL}} \right\}_{i}$ is the *i*th subset of the payload, $\varvec{A}_{i}$ is the *i*th array vector, *n* is the number of subsets in the payload.

In the checking phase, a positive membership is responded when all the cells addressed by a query string are 1 on a certain layer, while a negative membership indicates that there is at least one 0 in those addressed cells. A query string must go through all the parallel layers for checking its membership and corresponding class. Consequently, there are three types of result after the checking phase is completed, i.e., (a) no layer, (b) exactly one layer and (c) multiple layers are responded to the query string as positive membership. Situation (a) indicates that the query string is not a member of the payload at all. Since there is no false-negative error for PBF, the query string is definitely not a member. Situation (b) concludes that the query string is a member with a class of whatever the corresponding layer designates. However, such a situation could also be induced by non-member strings, which are referred to as false-positive errors. Situation (c) represents confusion, which could be caused by either a member or non-member.

### The proposed scheme

The CMAC paradigm was originally proposed for controlling robotic manipulators, which was later recognized as a neural network mapping because the paradigm also requires a weight-adjusting process by iteratively learning from a great amount of known data. We adopted the paradigm with modifications because of the following motivations: (1) the structure of CMAC, with hashing and a one-dimensional weight array, is very similar to Bloom filter, so the computational complexity for a query string is constant, (2) the learning process, although relatively time-consuming, is considerably faster than other neural network paradigms due to the locally weight-adjusting protocol, and (3) the paradigm could achieve substantially less errors than Bloom filter once the learning process is in order. Therefore, our modifications to the paradigm include: (1) a method referred to as “concatenation” in the original paradigm for the generalization purpose is disregarded because we only require the memorization capability and (2) a number of designated values in the output layer could be specified instead of one for the classification purpose with acceptance boundary of class membership in an attempt to reduce false-positive errors.

**Fig. 2:**
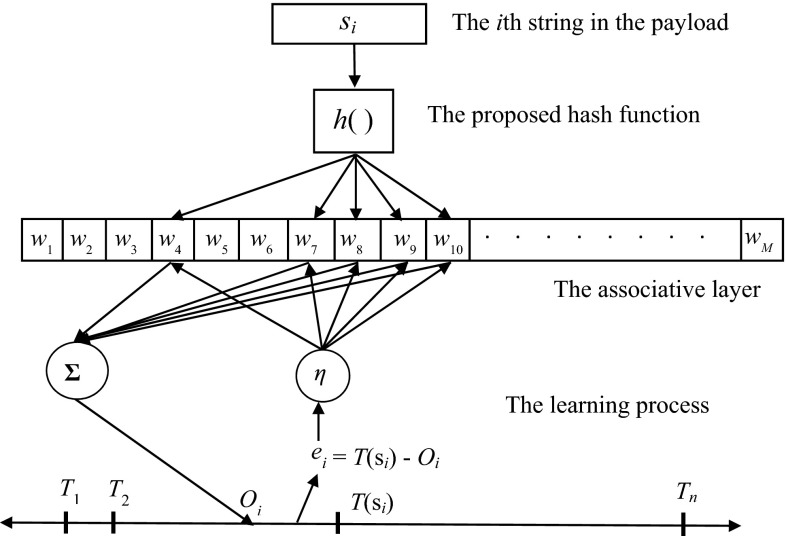
Learning process of the CMAC-based scheme

Since the proposed scheme comprises a single associative layer with cells of real numbers, classification could be achieved by assigning a different class code to a subset of the payload. The class codes are also referred to as targets in the scheme, representing the goal to achieve when adjusting the cell contents according to a string’s outcome resulted from the associative layer. Therefore, the learning phase is responsible for moving the outcomes of strings from the same subset toward its corresponding target. Like other neural network models, such a learning process is accomplished by modifying the cell values, also referred to as weights, in an iterative fashion according to the difference between outcome and target. Let *s*_*i*_ be the *i*th string in the payload, and the outcome of *s*_*i*_ could be described as in (5). Let *T*(*s*_*i*_) be the target value of *s*_*i*_, where *T*(*s*_*i*_) belongs to the set {*T*_*j*_, *j* = 1 ~ *n*} and *n* is the number of subsets or classes in the payload; modification of the cell values is described as in (6). The learning process, as depicted in Fig. 2, terminates when there is no significant cell value change within a number of iterations.

5 $$O_{i} = \mathop \sum \limits_{k = 1}^{{l\left( {s_{i} } \right)}} w(a(s_{i} \left( k \right)))$$

6 $$w\left( {a\left( {s_{i} } \right)} \right) = w\left( {a\left( {s_{i} } \right)} \right) + \eta \times \left( {T\left( {s_{i} } \right) - O_{i} } \right)$$

where *O*_*i*_ is the outcome of *s*_*i*_, *w* is the cell value and indexed by *w*(*k*) or *w*_*k*_, *a*(*s*_*i*_) is the set of cells addressed by *s*_*i*_, *η* is the fraction of difference between target and outcome, also referred to as learning rate.

The learning rate is a positive fractional number, representing the portion of error to be compensated back to the associative layer for the purpose of cell value adjustments. When $\eta = 1{/}l\left( {s_{i} } \right),$ the error is fully and evenly reflected to each of *a*(*s*_*i*_), which often causes an unstable learning process since normally each cell is addressed by a number of strings. In this paper, a smaller learning rate $\eta = 0.1{/}l\left( {s_{i} } \right)$ is used to avoid such a situation. After the learning phase is completed, another payload presentation is performed without cell value adjustments to store necessary information for the checking phase. There are two steps in this non-learning presentation: first, the number of memory bits to economically store each cell value must be decided for modifying all the weights in the associative layer. For example, when a 16-bit number is used for each cell, the cell values could be described as in (7) and (8).

7 $${\rm d}w = \left({w_{\rm max } - w_{\rm min}} \right)/\left( {2^{b} - 1} \right)$$

8 $$w_{i} = w_{\rm min } + {\text{round}}\left( {\frac{{w_{i} - w_{\rm min } }}{{{\rm d}w}}} \right)\,\times\,{\rm d}w$$

where d*w* is the weight increment when *b* bits of memory are used for each cell, *w*_max_ and *w*_min_ are the maximal and minimal weights after the learning phase is completed.

**Fig. 3:**
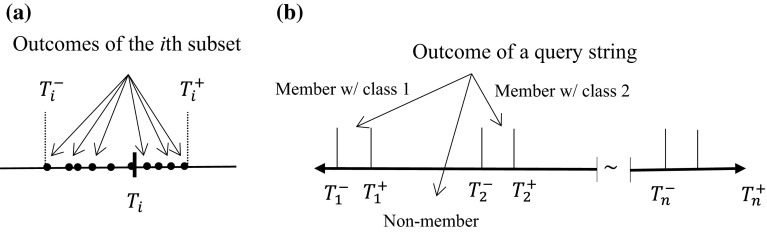
**a** Establishing boundaries of target, **b** determination results of the checking phase

Second, boundaries of membership must be established for each target, which is accomplished by calculating the minimal and maximal outcomes for each subset of the payload, designated by $T_{i}^{ - }$ and $T_{i}^{ + }$, *i* = 1 − *n*. Figure 3a shows the establishment for boundaries of membership. The boundaries of membership for all targets are also stored and passed to the checking phase for the on-line determination purpose. As shown in Fig. 3b, if the outcome of a query string is within the boundaries of any target, the string is a member with the corresponding class of that target; otherwise, the string is a non-member. It is clear that the results of the checking phase would not result in confusion error when there are no overlaps among the target boundaries, but false positives are still possible. The boundary overlap situation indicates that the outcomes of strings in the payload do not converge to their corresponding targets, which is normally caused by insufficient memory usage for the associative layer.

## Experimental results

To examine performances of the proposed scheme, we employed data of two simulation cases, including the car license plates in Taiwan and general-form email addresses. The former represents strings of the same length (seven characters including a separator), while the latter contains strings of varied lengths of between 10 and 25 characters. Table 1 shows string data examples for both simulation cases. The proposed hash function described in Sect. 2 was utilized as the sole hash function for both schemes, which produced the same number of bits as the length of the input string on the associative layer. Therefore, there were seven cells addressed by each string of the first case, while that of the second case differs from 10 to 25. We randomly generated 400,000 strings for each simulation case, in which 300,000 strings were used as the programming set, while the remaining 100,000 strings were utilized as the checking set.

**Table 1:** String examples of both simulation cases

Car license plate	Email address
OF-8543	k6tlh@wt.com
8753-3E	pia0@d55zg400djk.net
E5-4100	ngc2l@sia.org
6430-PE	xj5g@v7suzzme9au0.com
3879-UN	20x99@ouqh7qgg.org
2827-DM	ldqlk@58f.net
3228-OP	rc6nh@pi5y5woaf2y.com
9225-BA	tk6@th5iqeakorph.net

**Fig. 4:**
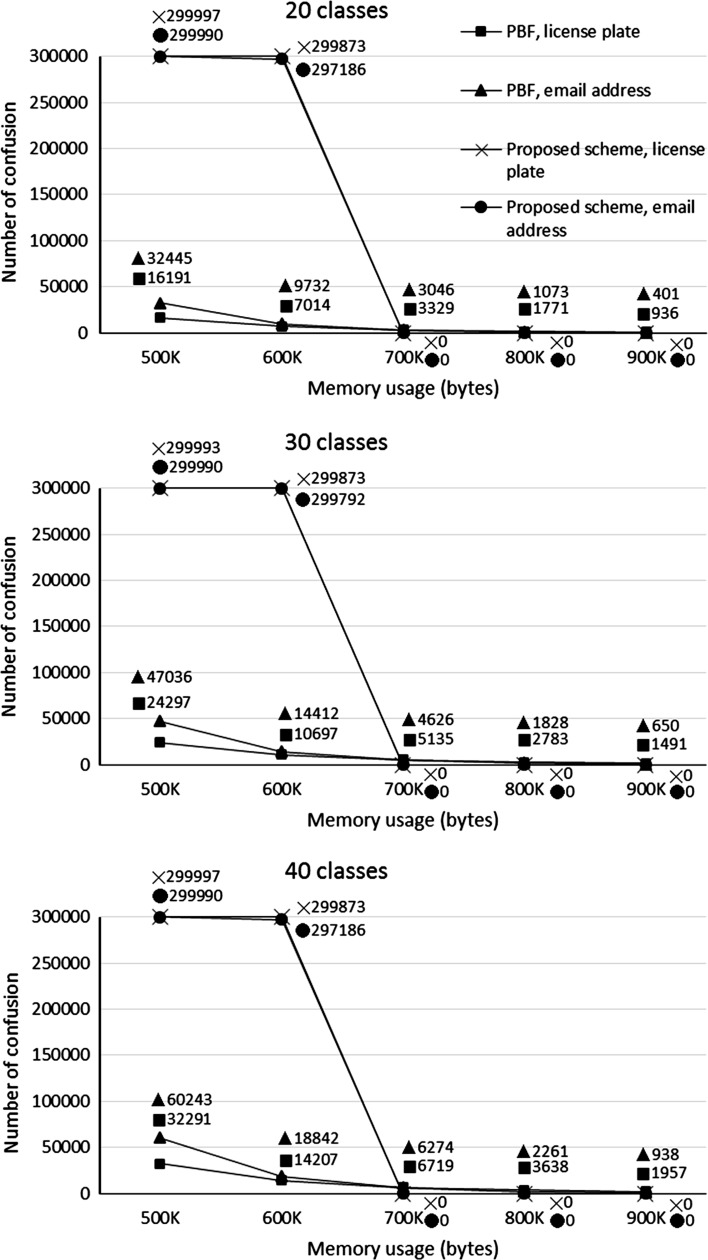
Numbers of confusion in the programming sets with varied memory usages

The programming sets were engaged in the first phase of both the PBF and the proposed scheme. Ideally, all the string data in the programming sets should be recognized as positive membership with a certain class after the first phase is completed; however, membership with multiple classes (i.e., confusion) could happen because of insufficient memory usage for the associative layer(s) in both schemes. Therefore, we conducted several experiments using varied memory usages in the first phase. Figure 4 shows the numbers of confusion in the programming sets after the first phase is completed, by which several observations could be obtained. First, as the memory usage increases from 500 to 900 KB, the number of confusion of PBF steadily decreases, while that of the proposed scheme reduces abruptly from almost the entire programming set to zero between 600 and 700 KB. This situation indicates that the boundaries of targets in the first phase of the proposed scheme mutually exclude one another when the memory space reaches 700 KB. Second, the number of confusion for PBF increases as the number of classes increases. This situation suggests that confusion of PBF is, to some degrees, related to the number of the associative layers, while that of the proposed scheme solely depends on whether there are overlapped sections among the boundaries of targets.

As seen in Fig. 4, after the neural network mapping using the proposed scheme, the string identifiers still with confusion accounted for almost the entire programming set for all the 20-, 30- and 40-class simulations. Such a situation was caused by insufficient memory utilized on the associative layer, i.e., <700 KB in our cases. The memory insufficiency usually leads to a deadlock condition where too many class targets compete one another in the same cells on the associative layer. Consequently, the target boundaries could not successfully converge to a mutually exclusive outcome. In other words, the majority of cells on the associative layer were addressed by a great amount of strings in the programming set, resulting in the weight values being unable to stabilize. However, as the memory usage reached 700 KB, all the target boundaries became mutually exclusive and there was no confusion whatsoever in the programming set.

The previous investigation concerning the number of confusion existed in the programming sets shows that when 700 KB of memory space was used, the ratio of confusion was zero for the proposed scheme or nearly zero (approximately 1 %) for PBF with both simulation cases. Therefore, we further investigated the number of errors in the checking sets committed by both schemes using the memory space starting with 700 KB for the associative layer. Since the checking sets are exclusive of the programming sets, there are two types of error using PBF, namely confusion and false positive, while only false-positive errors are possible using the proposed scheme since all the class boundaries do not overlap one another. The memory usage in the programming phase and the number of classes were the control variables in the experiments. The memory usage is a crucial factor when a scheme is used as a core component for real-time applications, which usually claims a good portion of the primary memory on the service machine. As far as PBF is concerned, the number of cells for each layer is the same, and so is the number of strings for each class in the programming sets. Consequently, when 700 KB of memory and 20 classes are specified, there are 700,000 bytes × 8/20 = 280,000 cells in each layer of PBF and 300,000/20 = 15,000 strings for each class in the programming sets. Table 2 presents the error numbers of both types, i.e., confusion and false positive, committed by PBF and the proposed scheme using different combinations of memory usage and number of classes.

**Table 2:** Simulation results in the checking phase

Simulation case	Memory usage	Error type	Number of strings with error in the checking set					
			20 classes		30 classes		40 classes	
			PBF	Proposed scheme	PBF	Proposed scheme	PBF	Proposed scheme
Car license plate	700 KB	C	3329	0	5027	0	6719	0
		FP	1174	222	1649	229	2273	365
	800 KB	C	1771	0	2783	0	3638	0
		FP	571	40	927	57	1156	81
	900 KB	C	936	0	1491	0	1957	0
		FP	336	8	546	15	661	17
	1 MB	C	579	0	861	0	1171	0
		FP	198	5	316	8	405	11
	1.1 MB	C	385	0	568	0	802	0
		FP	138	0	207	0	251	0
Email address	700 KB	C	3046	0	4688	0	6274	0
		FP	1042	312	1529	234	2099	268
	800 KB	C	1073	0	1686	0	2261	0
		FP	382	36	569	63	779	73
	900 KB	C	401	0	650	0	938	0
		FP	145	23	203	34	330	48
	1 MB	C	183	0	292	0	357	0
		FP	64	13	107	23	132	22
	1.1 MB	C	101	0	138	0	190	0
		FP	29	0	59	0	57	0

*C* confusion, *FP* false positive

As shown in Table 2, the number of errors decreases as the memory usage increases for both PBF and proposed scheme. Furthermore, the larger number the classes, the less the errors. The proposed scheme committed far less errors, approximately one twentieth, than the benchmark PBF with any combination of the two control variables, i.e., the memory usage and number of classes. The confusion errors of PBF account for approximately three-fourth of the total errors, which is the main cause of such results. Not only the proposed scheme committed no confusion error because of the non-overlapped boundaries among classes, but it became error-free when the memory usage reached 1.1 MB for the programming phase. That is to say, with a sufficient amount of memory space for the associative layer, the chance of a non-member string falsely passes through any membership boundaries for all classes is very rare.

In summary, there are two types of error, i.e., confusion and false positive, when dealing with the addressed problem. In the programming phase, the proposed scheme commits no confusion error when the memory is sufficient, while the benchmark still reports such errors with the same memory space. However, the proposed scheme could hardly recognize any membership when memory is inadequate, while the benchmark could still recall roughly 80–90 % of membership depending on the size of memory. In the checking phase, the error rates of both types committed by the proposed scheme were much less than the benchmark. As far as time is concerned, the proposed scheme requires inevitably more time because of the neural network mapping, approximately 2000 presentations of the dataset, in the programming phase, while the benchmark only needs one presentation. In the check phase, the time required for processing a query string is instant for both schemes since they have the identical computational complexity for one pass.

## Conclusion

We present a scheme employing a neural network mapping for simultaneously determining the membership and classification for string identifiers. The objective of the proposed scheme is to achieve prompt responses, indicating membership of a query string and its associative attributes at the same time. Furthermore, the memory usage at the run time must be as economical as possible because it normally occupies the primary memory of the service machine. Such a requirement becomes crucial when the number of strings in the payload is tremendous, which is often conceivable as the Internet is being expanded at a very fast speed. The experimental results show that the proposed scheme outperformed the benchmark, the PBFs, as far as the numbers of error committed are concerned with the same combinations of control variables. The results are based on several scales of the control variables including the memory usage and number of classes in the payload. As a result, the proposed scheme committed less number of misjudgments than the benchmark in any combination of the control variables.

The results suggested that the proposed scheme seems to be a promising approach when economical memory and accuracy in checking membership with classification are of concern. Although the proposed scheme is associated with a cost that certain amounts of computational time are required in the learning phase. In our experiments, however, no more than 2000 iterations of payload presentation were performed for all the combinations of control variables. Such a cost could become small when on-line checking speed and memory usage are critical measures of performance, since the learning phase usually takes place in an off-line mode. As far as the test results are concerned, the benchmark still committed a fairly large number of confusion error even sufficient memory is employed, while the proposed scheme is immune from such error. Although both schemes inevitably associate with false-positive errors, the proposed scheme committed less false rates than the benchmark in all aspects. It is conceivable that there will be no prediction errors for both the proposed scheme and the benchmark when the memory is unlimited. However, the memory is usually constrained and related to the number of string identifiers in the payload, e.g., 300,000 for both simulation cases in our experiments. Future work will focus on exploring rules for determining the optimal memory usage with respect to the payload size including the number and length of the string identifiers.
